# Gut Microbiota-Mediated Modulation of Intestinal Serotonin by Tea Polyphenols: Potential Mechanisms and Implications for Metabolic Health

**DOI:** 10.3390/nu18101606

**Published:** 2026-05-18

**Authors:** Lili Jiang, Yonghui Yu, Min Zheng, Xinyu Cao, Juxiu Li

**Affiliations:** 1 College of Food Science and Engineering, Northwest A&F University, Yangling 712100, China; lilijiang2025@nwafu.edu.cn (L.J.); zm152952@163.com (M.Z.); 18238600227@163.com (X.C.); 2 Hainan Institute, Northwest A&F University, Sanya 572025, China; 3 Key Laboratory of Geriatric Nutrition and Health, Ministry of Education, Beijing Technology & Business University, Beijing 100048, China; yonghuiwh@btbu.edu.cn

**Keywords:** tea polyphenols, gut microbiota, serotonin, gut homeostasis, metabolic disorders

## Abstract

Tea polyphenols (TPs), the primary bioactive components derived from tea, play an important role in maintaining gut function and metabolic homeostasis. Emerging evidence indicates that the regulatory functions of TP within the intestine are intricately linked to their interactions with the gut microbiota. Serotonin (5-hydroxytryptamine, 5-HT), a key signaling molecule in the gastrointestinal tract, has been implicated in preserving intestinal function and metabolic health. Notably, the modulation of gut microbiota by TPs, the microbial biotransformation of TPs into bioactive metabolites, and the potential regulation on intestinal 5-HT homeostasis may collectively constitute an interconnected axis relevant to gut health and metabolic balance. However, direct experimental evidence linking these components into a unified mechanistic pathway remains limited, and the molecular basis of this putative TP–microbiota–5-HT axis requires further validation. This review systematically summarizes and discusses the regulatory effects of TPs on gut microbiota, and the microbial biotransformation of TPs into metabolites, as well as the microbial modulation of intestinal 5-HT and the roles of 5-HT and its receptors in intestinal function and homeostasis, with a particular focus on critically evaluating the extent to which current data support the proposed interactions among TPs, gut microbiota, and 5-HT in metabolic disorders. A deeper understanding of this tripartite interaction may ultimately inform the development of TP-based dietary approaches targeting gut microbiota–5-HT interactions in gut function and metabolic health, although such translational applications remain speculative in the absence of robust causal evidence.

## 1. Introduction

Tea, one of the most widely consumed beverages globally, owes its health-promoting properties primarily to its rich polyphenolic content [1,2]. Tea polyphenols (TPs) constitute a chemically heterogeneous group of phytochemicals, mainly comprising catechins. The major catechins derivatives include (−)-epigallocatechin-3-gallate (EGCG), which accounts for 50–80% of total catechins, as well as (−)-epicatechin (EC), (−)-epigallocatechin (EGC), and (−)-epicatechin-3-gallate (ECG). A growing number of studies have demonstrated that TPs and their principal bioactive constituents exhibit a wide array of biological activities, including antioxidant, anti-inflammatory, and metabolism-modulating effects [3,4,5], with notable beneficial effects in the context of diet-related metabolic disorders such as obesity. However, the low bioavailability of TP indicates that its intestinal health effects are profoundly dependent on its bidirectional interaction with the gut microbiota, as a significant portion of ingested TPs reaches the colon unabsorbed [6,7]. Experimental evidence indicates that TPs can reshape the gut microbial structure by directly inhibiting pathogens and indirectly promoting beneficial bacteria, such as Akkermansia and Lactobacillus, thereby stimulating the production of short-chain fatty acids (SCFAs) [3,8]. On the other hand, the gut microbiota metabolizes TP into more bioactive small molecules through its unique enzymatic repertoire [9]. Collectively, these interactions improve the luminal redox state, inflammatory response, and intestinal barrier, thereby modulating intestinal health and metabolic homeostasis [10,11].

As the predominant signaling molecule in the gut, over 90% of the body’s serotonin (5-hydroxytryptamine, 5-HT) is produced by enterochromaffin (EC) cells. 5-HT signaling homeostasis is thought to play an important part in coordinating motility, secretion, and barrier function, and is closely related to functional gastrointestinal disorders and inflammatory bowel disease [12]. Notably, the favorable microenvironment and specific metabolites including SCFAs generated from this TP–microbiota crosstalk have been proposed as potential regulators of intestinal 5-HT synthesis and release [13,14]. Emerging evidence further suggests a possible association between dysregulation of the gut microbiota–5-HT axis and metabolic disturbances, particularly high-fat diet (HFD)-induced obesity and related metabolic disorders [15]. Therefore, it is plausible but not yet established that TP may influence gut 5-HT homeostasis through microbiota-dependent mechanisms and that the microbiota–5-HT interaction could contribute to the effects of TP on intestinal homeostasis and metabolic health. However, direct evidence linking TP-induced gut microbial changes to altered 5-HT signaling and subsequent metabolic outcomes remains limited. This review aims to summarize the current evidence on the interplay among TP, gut microbiota, and 5-HT signaling, and clarify the extent to which existing data supports direct mechanistic links versus correlative or hypothesized associations. We performed a structured search of the literature using PubMed, Web of Science, and Scopus from 1996 to 2026. We used combinations of key search terms including (“tea polyphenols” OR “green tea catechins” OR “EGCG”) AND (“gut microbiota” OR “microbial metabolites”) AND (“serotonin” OR “5-HT” OR “5-HTRs”), supplemented with related terms such as “intestinal barrier,” “obesity, “glucose metabolism,”lipid metabolism and “metabolic health.” Peer-reviewed original research articles and authoritative reviews were prioritized. For the background related aspects of 5-HT biology, highly cited and foundational papers published before 2016 were included. For mechanism analysis and discussions on the TP–microbiota–5-HT axis, we focused on primary studies published within the last ten years (2016–2026).

## 2. TP Reshape Gut Microbiota

**Figure 1:**
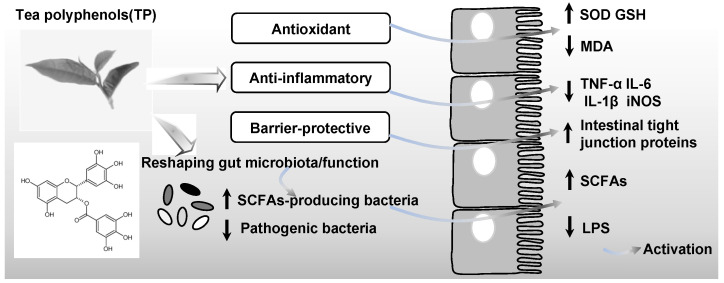
Effects of tea polyphenols on the gut microbiota and the maintenance of host homeostasis. TP intervention promotes the expansion of short-chain fatty acid (SCFA)-producing bacteria while suppressing pathogenic bacteria, leading to increased levels of SCFAs and reduced release of lipopolysaccharide (LPS). This functional shift in the gut microbiota leads to an anti-inflammatory, antioxidant, and barrier-protective (e.g., enhanced intestinal tight junction proteins) environment within the gut, collectively contributing to improved host metabolic health. Arrows denote upward (↑) and downward (↓) trends.

To date, it has been confirmed that TP improve gut microbiota structure mainly via direct antimicrobial actions and indirect probiotic-like effects (Figure 1). TP exhibit direct effects by selectively inhibiting the growth of specific pathogens and opportunistic bacteria [16]. For instance, TP can inhibit biofilms formation by the opportunistic pathogen *Candida albicans* [17]. The indirect effects of TPs mainly involve their ability to improve the intestinal microenvironment. TPs enhance the survival and colonization of beneficial obligate anaerobes, not as conventional prebiotics, but by scavenging luminal reactive oxygen species and relieving oxidative stress [18,19]. Additionally, TPs suppress gut inflammation by reducing pro-inflammatory cytokines (including TNF-α and IL-6) via inhibiting inflammatory signaling pathways, such as nuclear factor kappa-B (NF-κB), which helps preserve both the intestinal barrier and the structure of the microbial community [3].

### 2.1. Modulation of Microbial Composition Toward a Beneficial Profile

Mounting evidence suggests that TPs play an important role in regulating the gut microbial ecosystem. In general, TPs reshape the gut microbiota toward a beneficial phenotype by enriching beneficial commensals and suppressing pathogens, thereby enhancing the production of microbial metabolites such as SCFAs and maintaining intestinal homeostasis. EGCG and Liubao insect TPs have been shown to mitigate intestinal inflammation and improve the intestinal barrier by enriching SCFA-producing bacteria such as *Akkermansia*, but inhibiting the harmful bacteria *Turicibacter* and *Erysipelatoclostridium* [3,8], which consequently promotes SCFA generation. TPs from green tea (containing 49.27% EGCG, 10.09% ECG, 15.49% EGC, and 7.18% EC) also restore gut microbial richness and diversity impaired by antibiotic treatment and enhance the abundance of beneficial taxa such as *Lactobacillus*, *Akkermansia*, and *Blautia* [20]. In obese mice, TPs mitigate obesity-related inflammation by increasing *Blautia* and *Faecalibaculum* and reducing *Colidextribacter* abundance; meanwhile, TPs upregulate the expression of intestinal tight junctions and suppress inflammatory cytokine production as well as toll-like receptor 4 (TLR4) signaling pathways, thereby preserving intestinal barrier integrity [21]. Furthermore, TPs (e.g., EGCG) reduce serum lipopolysaccharide (LPS) translocation by upregulating the relative abundance of *Lactobacillus*, *Muribaculaceae* and *Clostridia*_UCG-014 and downregulating *Desulfovibrio*, enhancing the generation of SCFAs [22,23]. Consistently, multiple reports have confirmed that polyphenol-rich Oolong TPs and EGCG interventions restore microbial diversity and remodel the gut microbiota composition [24,25]. A multi-omics pilot study in healthy adults showed that Oolong TP consumption significantly altered gut microbial diversity (Shannon index), and enriched Bacteroides and *Prevotella* [4]. In HFD-induced obesity mice, green TP increased the richness and diversity of colonic microbiota and acetic and butyric acid levels in a dose-dependent manner [26]. The distinct microbial taxa enriched by TPs likely arise from variations in their chemical composition, origin, intervention duration, and host physiological status.

Recent results have shown that EGCG normalized intestinal microbial dysbiosis in a mouse model of metabolic disorders treated with bisphenol-A [27]. A comparative study demonstrated that raw TPs and ripened TPs differentially regulate the enterotypes of HFD-treated mice to restore intestinal microbiota dysbiosis [28]. TPs (including EGCG) reduce fat accumulation and systemic inflammation partially by improving the intestinal microbiota composition, and these microbial alterations are remarkably associated with bile acid metabolism disorders in obese mice [29,30]. However, the precise mechanisms linking TP-regulated bile acid-metabolizing microbiota to gut metabolic homeostasis remain limited and merit further in-depth investigation.

### 2.2. Improvement of Gut Luminal Environment Through Antioxidant and Barrier-Protective Effects

The chemical structural features of TPs are closely related to their notable antioxidant potential [5], which further facilitates the gut microbial microenvironment. This is the fundamental mechanism by which TPs reshape the intestinal microbiota and indirectly maintain intestinal homeostasis. TPs act as direct ROS scavengers and directly increase the activities of antioxidant enzymes, including superoxide dismutase and catalase, and immune enzymes such as acid phosphatase, which collectively improve the antioxidant capacity and immune response, accompanied by a marked reduction in potential pathogens, especially *Clostridiaceae* [31]. TPs modulate gut microbiota composition in a dose-dependent manner by maintaining intestinal redox balance, though excessive intake diminishes their beneficial effects [10]. Liubao insect TPs prevent gastric injury through enhancing antioxidant defenses (elevating superoxide dismutase while suppressing malondialdehyde and myeloperoxidase) and attenuating inflammation (downregulating TNF-α, IL-6, IL-1β, and iNOS), thereby preserving mucosal integrity [32]. In squabs, TPs reinforce intestinal antioxidant defenses primarily by activating the nuclear factor erythroid 2-related factor 2/antioxidant response element pathway, an effect closely associated with the modulation of the ileal microbiota, including increased abundance of *Corynebacterium*, which enhances redox homeostasis and shapes a healthier gut luminal environment [33]. A clinical trial in male athletes further demonstrated that green TPs significantly reduced systemic oxidative stress (lower malondialdehyde and higher total antioxidant capacity), underscoring the systemic antioxidant capacity of TP, which indirectly support gut luminal redox balance and barrier integrity [34]. In addition, the antioxidant ability of Oolong TP helps maintain intestinal barrier integrity and suppress the endotoxins (e.g., LPS)) translocation, thus lowering systemic low-grade inflammation [22,35]. Catechin-rich TPs upregulate the expression of intestinal tight junction proteins, depress the expression of TLR4/NF-κB-dependent inflammatory genes, as well as increase gut microbial diversity [36]. Moreover, recent studies highlight the role of tryptophan metabolites in this process. Gut microbiota-derived indole metabolite indole-3-acetic acid mediates the protective effects of Fu brick TPs on intestinal barrier improvement and colitis relief via the aryl hydrocarbon receptor signaling pathway [37]. This suggests that TP-based microbial metabolism of precursors (like tryptophan) into bioactive ligands like indoles is a crucial layer of regulation.

Notably, the modulatory efficacy of TP on gut microbial composition and metabolism is not isolated but shaped by interactions with other co-existing dietary components in the intestinal lumen. TP and arabinoxylan oligosaccharides both improve metabolic disturbances in diet-induced obesity, but exhibit opposing effects on the gut microbiota. Specifically, arabinoxylan oligosaccharides enrich *Bifidobacterium* and reduce α-diversity, whereas green TP reverses these shifts in a dose-dependent manner [38]. These changes illustrate that the ability of TP to shape the luminal microenvironment is context-dependent and influenced by concurrent dietary components and the existing microbial landscape.

## 3. Biotransformation of Tea Polyphenols by Gut Microbes and Their Bioactivity Metabolites

Owing to their high molecular weight and structural complexity, the majority of TPs are poorly absorbed in the small intestine. Therefore, the gut microbiota is important in the digestion, absorption, and utilization of TP, which is achieved by the participation of a cascade of exclusive gut microbial enzymes that chemically modify TPs, influencing both their bioactivity and bioavailability in the host. Glycosidases catalyze the hydrolysis of glycosidic linkages in flavonoids to generate bioactive aglycone moieties. Galloyl esterases specifically target ester bonds, such as the gallate ester in (−)-epigallocatechin-3-gallate and carbon-carbon lyases mediate the cleavage of the catechin C-ring [9]. Substantial inter-strain variability exists in both the repertoire and expression levels of genes encoding these enzymes, resulting in marked differences in the capacity of gut microbes to metabolize TP. This functional heterogeneity is exemplified by studies showing that only specific taxa, such as *Adlercreutzia*, *Eggerthella*, and *Lactiplantibacillus plantarum*, catalyze key biotransformations of tea catechins, including C-ring cleavage, degalloylation, and deglycosylation, yielding derivatives with enhanced antioxidant activity. Concurrently, TPs and their microbial metabolites exert broad inhibitory effects on members of Actinobacteria, *Bacteroidetes*, and *Firmicutes*, while *Lactobacillus* species remain relatively unaffected [39]. This finding further underscores the metabolism-dependent selectivity rooted in microbial genomic and enzymatic diversity.

Following this transformation, the biological activity of TPs can be increased. The human small intestine cannot fully absorb TP. The majority of ingested TPs reach the large intestine, where they are metabolized and converted by the gut microbiota into various bioactive derivatives. In a rat model, gallate ester in (−)-epigallocatechin-3-gallate underwent microbial ring fission as the principal metabolic transformation, generating 4-hydroxy-5-(3,5-dihydroxyphenyl) valeric acid as the major microbial metabolite [6]. The primary degradation pathways of tea catechins include degalloylation, C-ring cleavage, and A-ring fission. Microbial esterases, which are enzymes that broadly distributed among gut bacterial species, catalyze the hydrolysis of the ester bond linking gallic acid to the flavan-3-ol backbone [7]. Ibetan TP resists upper gastrointestinal digestion but is extensively fermented in the colon, which differentially enriches beneficial bacteria such as Bifidobacterium, Faecalibacterium, and butyrate-producing bacteria and markedly enhances SCFA production, thereby exhibiting prebiotic potential [40]. While the gut microbiota metabolizes TPs into bioactive derivatives, it also modulates the chemical fate of polyphenols in the presence of dietary toxicants. One study demonstrated that a complex gut microbial community completely suppressed the formation of potentially harmful quinone products generated from the reaction between TPs and N-nitrosamines, suggesting a protective role of the microbiota beyond direct metabolism [41]. The various bioactive small-molecule metabolites produced after TPs are metabolized by gut microbiota, including phenolic acids (such as 4-hydroxybenzoic acid and 3-methoxy-4-hydroxyphenylacetic acid) and valerolactones, have a wide range of physiological functions and play a key role in regulating intestinal function and homeostasis. These metabolites exhibit antioxidant, anti-inflammatory properties and metabolic regulatory effects, which help prevent intestinal oxidative injury, reduce systemic inflammation, and thereby maintain intestinal function and metabolic health [11,42].

Furthermore, TP aggregates (macromolecular complexes formed during microbial fermentation) are composed of flavan-3-ol backbones with flavonol/anthocyanin termini and integrated phenolic acids and amino acids. A recent study demonstrated that they exert anti-inflammatory effects by suppressing TLR4/myeloid differentiation primary response gene 88-NF-κB signaling, thereby reducing pro-inflammatory mediators in vitro by ameliorating colitis in vivo through preserving intestinal barrier integrity, modulating systemic inflammation, and reshaping the gut microbiota, highlighting their role as key microbial bioactive mediators of TP in the modulation of the gut–immune system axis [43].

## 4. Effects of Intestinal 5-HT in Maintaining Gut Motility, Secretion, and Barrier Integrity

### 4.1. Biosynthesis and Microbial Regulation of Intestinal 5-HT

The gastrointestinal (GI) tract is the primary site for the synthesis of 5-HT, with more than 90–95% of the total body 5-HT being generated in this organ. This process is mainly dependent on EC cells, which are specialized endocrine cells scattered throughout the GI tract [44,45]. The biosynthesis of 5-HT begins with dietary intake of the essential amino acid tryptophan. After absorption in the small intestine, tryptophan enters the bloodstream and is subsequently taken up by EC cells. Within these cells, tryptophan is first catalyzed by the key rate-limiting enzyme tryptophan hydroxylase 1 (TPH1) into 5-hydroxytryptophan (5-HTP), which is then rapidly decarboxylated by aromatic L-amino acid decarboxylase to produce 5-HT [46]. Concurrently, the removal of intestinal 5-HT is largely regulated by the gut luminal microenvironment. This involves the reuptake mechanism mediated by the membrane-associated serotonin reuptake transporter (SERT) and the subsequent catabolism within enterocytes [47]. It is important to note that TPH1 is expressed exclusively in peripheral tissues, such as the gut, whereas the central nervous system relies on the isoform TPH2 for 5-HT synthesis, ensuring functional independence from each other due to the blood–brain barrier.

Despite being synthesized entirely by host EC cells, host 5-HT systems are extensively modulated by the gut microbiota and its diverse metabolites through directly and indirectly regulatory manners [48]. Germ-free mice exhibit lower levels of 5-HT and decreased colonic contractile duration, highlighting the main regulatory role of gut microbiota in maintaining intestinal 5-HT generation and normal gut motility [49]. A variety of probiotics and their active products have been found to participate in this process. The probiotics *Akkermansia muciniphila* was found to activate the 5-HT signaling in intestinal epithelial cells, and its secreted extracellular vesicles further mediate colonic 5-HT production [50,51]. Among microbial metabolites, SCFAs are the most well-reported regulators. SCFA supplementation can enhance TPH1 transcription in human EC cell model BON cells [13]. *Lactobacillus paracasei* promotes colonic 5-HT production by modulating the gut microbiota to increase acetic acid concentration, which stimulates EC cells [52]. In piglet models, *Lactobacillus* and its microbial metabolites, including acetate, were shown to promote TPH1 expression and 5-HT production in IPEC-J2 cells through free fatty acid receptor 3 (FFAR3) [53], while in mouse models, *Bifidobacterium dentium* and its acetate metabolite stimulated 5-HT release from EC cells by activating FFAR2 [54]. This FFAR-dependent regulatory pattern reveals species-specific heterogeneity across human, porcine, and murine models, underscoring the importance of rigorous translational assessment of preclinical data prior to human applications.

Except for SCFAs, other microbial metabolites and signaling molecules constitute a complex regulatory network for intestinal 5-HT. Gut bacteria metabolize 5-HTP to 5-hydroxyindol and facilitate 5-HT release [55]. Indole and indole-3-carboxyaldhyde, typical tryptophan of catabolites of gut microbiota, indirectly induce 5-HT release from entero-endocrine cells in both mouse and human small intestine [14]. Additionally, recent findings have indicated that gut microbiota-derived bile acids stimulate the secretion of 5-HT, and this effect may be mediated by the G protein-coupled bile acid receptor 1 [56,57]. Among the microbial-derived metabolites examined, tryptamine and tyramine also promote 5-HT in the gut [13,58]. Thus, gut microbiota acting via SCFAs and indoles are crucial factors of enteric 5-HT generation and its homeostasis. Of note, genetically engineered *Escherichia coli Nissle* capable of producing 5-HT in situ provides direct evidence that microbiota-derived 5-HT can functionally modulate host intestinal motility [59,60]. Specific human-derived gut lactobacilli, namely *Limosilactobacillus mucosae* and *Ligilactobacillus ruminis*, can directly convert 5-HTP to 5-HT, thereby increasing colonic serotonergic innervation [61]. In addition, conditioned medium from *Limosilactobacillus reuteri* stimulates the release of 5-HT and other enteroendocrine hormones from an engineered jejunal organoid line [62].

It is noteworthy that EC cells integrate diverse luminal cues through a variety of nutritional sensing receptors [12]. In addition to FFAR, EC cells express olfactory receptors, G protein-coupled bile acid receptor 1, GPR142, and GPR119, among others. These receptors are important for the release of 5-HT, independent of TPH1-mediated synthesis. This sensory complexity enables the gut to finely regulate 5-HT output in response to a wide range of environmental stimuli, while maintaining overall functional homeostasis [14,63]. EC cells also function as chemo-sensors, expressing transient receptor potential ankyrin 1, transient receptor potential vanilloid 1. In addition to chemosensory receptors, EC cells express mechanosensitive piezo-type mechanosensitive ion channel component (Piezo1/2) that respond to luminal distension. Preliminary evidence suggests that pharmacological inhibition of Piezo channels upregulates TPH1 expression and enhances 5-HT release, suggesting mechanical cues may serve as a potential modulator of 5-HT homeostasis [64]. However, the physiological significance of this pathway in basal gut motility remains to be fully elucidated. Other receptors like the activation trace amine-associated receptor on EC cells can drive 5-HT release, thereby accelerating colonic transit and fluid secretion [65].

### 4.2. 5-HT-Mediated Regulation of Gut Motility and Secretion

Following release from EC cells, intestinal 5-HT primarily exerts biological functions through binding to specific 5-HT receptors (5-HTRs) to orchestrate gastrointestinal functions. To date, more than 15 different 5-HTRs subtypes have been characterized, among which five receptor families were observed in the intestine: 5-HTR1, 5-HTR2, 5-HTR3, 5-HTR4, and 5-HTR7. Except for the ionotropic 5-HTR3, all intestinal 5-HTRs families are G-protein-coupled receptors [44]. Different 5-HTRs families and their subtypes are distributed across various cell types along the digestive tract and mediate distinct physiological functions.

A study demonstrated that 5-HTR1 is expressed in human colonic epithelial Caco-2 cells [51]. Specifically, members of the 5-HTR1 family, such as 5-HTR1A and 5-HTR1P, have been identified in enteric neurons, mediating either inhibitory or excitatory effects in a subtype-dependent manner. Recent studies suggest that the 5-HTR1P exists in human small submucous neurons and the large intestine [66]. Activation of the 5-HT1A suppresses the release of neurotransmitters into intestinal motor neurons and perturbs motility by reducing colonic migratory motor complexes [67]. In contrast, activation of 5-HTR1P exerts an excitatory effect by triggering the intestinal peristaltic reflex and inducing chloride secretion in the gut [68,69].

The 5-HTR2 families comprises three main subtypes, including 5-HTR2A, 5-HTR2B and 5-HTR2C, and 5-HTR2B is crucial for normal gut motility [70]. Among them, the 5-HTR2B is the key subtype responsible for normal GI motility. Ex vivo trials have indicated that the excitatory effects of 5-HT are partially mediated by 5-HTR2B in both rodent and human intestines [71]. Activation of 5-HTR2B facilitates the development of enteric neurons and the proliferation of interstitial cells of Cajal, which play an important role in peristaltic movements [72].

Unlike other 5-HTRs expressed in the GI tract, 5-HTR3 functions as ligand-gated ion channel, permitting the influx of cations, including Ca^2+^ [73]. 5-HTR3 is expressed in intrinsic and extrinsic sensory afferents, and is widely distributed throughout the human colon and ileum [74,75]. RNA-seq analysis has revealed that 5-HTR3 is significantly higher in the distal colon than in the proximal colon [76]. Notably, the activation of 5-HTR3 on mucosa-innervating extrinsic sensory neurons requires exposure to 5-HT at micromolar concentrations [76]. Functionally, activation of 5-HTR3 stimulates enteric neurons to evoke peristaltic contractions and promotes chloride and fluid secretion [77,78,79,80]. Consistently, the 5-HTR3 antagonist ondansetron abolishes spontaneous contractions and attenuates peristaltic responses evoked by mucosal stimulation in isolated colonic preparations [81]. Altogether, these findings highlight the critical role of 5-HTR3 in the regulation of intestinal motility and secretion [82].

5-HTR4 is a G-protein-coupled receptor widely present throughout the GI tract. In the colon epithelium, 5-HTR4 mediates chloride secretion, particularly in the proximal colon [74,83,84]. Genetic and pharmacological studies in mice have demonstrated that physiological 5-HTR4 expression is important for maintaining normal colonic transit and defecation function [85,86]. Within the enteric nervous system (ENS), activation of 5-HTR4 elicits peristaltic reflexes by acting on presynaptic nerve terminals to promote the release of excitatory neurotransmitters, such as acetylcholine [87,88]. Stimulation of 5-HTR4 on epithelial cells also promotes Cl^(−)^ secretion and GI motility [83,84]. Accordingly, selective 5-HTR4 agonism has been shown to increase gastric contractile amplitude and accelerate gastric emptying, highlighting its physiological importance in maintaining normal gastrointestinal transit [89]. In rats with diabetes mellitus, the agonist prucalopride of 5-HTR4 improves intestinal motility by increasing ENS regeneration [90]. In addition, the expression of 5-HTR4 is also subjected to post-transcriptional regulation by miRNAs miR-16 and miR-103 [91].

5-HTR7 is expressed in enteric neurons that elicit slow depolarization in response to 5-HT stimulation [92,93]. Previous results have suggested that 5-HTR7 is present in enteric neurons and smooth muscle cells of the guinea pig ileum, the entire digestive tract of the rat, as well as the human ileum and colon [74,93,94]. In the guinea pig ileum, 5-HTR7 activation mediates peristalsis and modulates circular muscle tone [93]. Notably, emerging evidence suggests that other 5-HT receptor subtypes (e.g., 5-HTR6) may also participate in the regulation of intestinal motility. However, the peripheral expression, physiological functions and therapeutic potential of 5-HTR6 in gastrointestinal disorders remain unclear [95].

### 4.3. 5-HT Homeostasis and Maintenance of Intestinal Barrier Function

Beyond its well-reported roles in regulating intestinal motility and secretion, serotonergic signaling plays a critical role in modulating intestinal mucosal integrity and barrier function. Given the close association between disruptions in 5-HT homeostasis and intestinal inflammatory and stress-related gut disorders, it is vital to understand how 5-HT modulates barrier function under both physiological and pathological conditions to enhance intestinal health in mammals and food-producing animals.

Regarding barrier integrity, the role of 5-HT is dual and context-dependent, because it can be helpful for maintaining epithelial homeostasis and facilitating repair, yet it can also contribute to barrier dysfunction and exacerbate inflammatory responses under specific pathological conditions. In constipation models, specific *Lactobacillus* strains were found to enhance secondary bile acid formation, which activates G protein-coupled bile acid receptor 1 on EC cells, upregulates TPH1 expression, and increases 5-HT release, thereby stimulating goblet cell mucin secretion [57]. Additionally, the supernatant of *Lactobacillus* has been shown to increase colonic 5-HTR4 expression, thereby enhancing MUC2 secretion and improving barrier function in constipation mouse models, while also favorably modulating the gut microbiota [85]. Activation of 5-HTR4 induces mucosal 5-HT secretion and goblet cell degranulation [83]. Beyond increasing mucin secretion, gut-derived 5-HT is important for maintaining intestinal barrier integrity by regulating epithelial turnover, crypt architecture, and mucosal metabolism. This is evidenced by the fact that a deficiency of 5-HT leads to crypt hyperplasia, dysregulation of stem cell proliferation, and disruption of the colonic microenvironment [96]. Contrary to the protective roles, accumulating evidence indicates that excessive or dysregulated 5-HT signaling may be detrimental to barrier integrity and promote inflammation. For instance, mice with a knockout of TPH1 exhibited significantly reduced 5-HT production, attenuated inflammatory responses, and upregulated β-defensin levels. Furthermore, 5-HT inhibited β-defensin production in HT-29 cells [97]. It was recently found that *Akkermansia muciniphila*-derived extracellular vesicles can repair intestinal barrier integrity via enriching the beneficial *Bifidobacterium*, suppressing pathogenic *Mucispirillum*, and reducing colonic 5-HT overproduction in a dextran sulfate sodium-induced colitis mouse model [50]. Additionally, suppression of 5-HT synthesis mitigated the stress-induced diarrhea response and upregulated intestinal Occludin and Claudin-1 expression [98]. Moreover, a growing number of evidence from livestock models has also verified the close relationship between the 5-HT signaling system and intestinal barrier integrity under stress conditions. In an LPS-induced immune stress model of broiler chickens, lower levels of intestinal 5-HT synthesis and decreased expression of 5-HTR1 in the ileum were associated with impaired intestinal mucosal barrier function [99]. Similarly, in weaned stress piglet models, the expression levels of 5-HTRs were closely correlated with intestinal inflammatory responses and immune status [53], further suggesting that 5-HT signaling is involved in stress-induced intestinal dysfunction in livestock. This contradiction highlights the complexity of serotonergic signaling, which depends on the 5-HTRs subtypes and the pathological condition.

Additionally, 5-HT signaling homeostasis is crucial for gut motility-related gut dysfunctions, particularly constipation. For instance, previous results found that decreased production of 5-HT from enteric neurons leads to defects in ENS development and GI motility. However, the use of slow-release 5-HTP in mice restored 5-HT to the ENS and normalized GI motility and the growth of the enteric epithelium [100]. Given their importance in modulating GI functions, several therapeutic targets involving 5-HT-related proteins have been developed or repurposed to address motility disorders. Luminal administration of 5-HTR4 agonists increases colonic motility; conversely, luminal administration of 5-HTR4 antagonists reduces colonic motility [101]. Gut Piezo1 is essential for systemic 5-HT synthesis [63]. In a loperamide-induced functional constipation mouse model, dual deficiency of Piezo1/2 exacerbates intestinal dysmotility, structural abnormalities, and downregulation of key mediators such as substance P and 5-HTR3, highlighting their cooperative role in maintaining intestinal function through EC cell-mediated serotonergic signaling [102]. Collectively, 5-HT functions as a master regulator of intestinal homeostasis by coordinately modulating motility, secretion, and barrier function through receptor-specific signaling pathways, and plays a critical role in the pathophysiological resolution of gut function disorders.

## 5. The Gut Microbiota–5-HT Signaling in Metabolic Health and Its Regulation by Tea Polyphenols

### 5.1. Gut Microbiota–5-HT Signaling in the Regulation of Metabolic Disorders

Intestinal-derived 5-HT plays an important role in the pathogenesis of metabolic dysfunction, including obesity, diabetes and dyslipidemia [103,104], and its synthesis and signaling are tightly regulated by the gut microbiota. Dysregulated 5-HT signaling was shown to exacerbate HFD-caused obesity and insulin resistance in mice [103]. Mechanism studies reveal that peripheral 5-HT exacerbates postprandial dyslipidemia associated with type 2 diabetes and obesity by promoting dietary fat absorption and chylomicron secretion via intestinal 5-HTR4 activation [105]. Meanwhile, 5-HTR4 antagonist GR113808 suppressed body weight gain and improved glucose intolerance in HFD-fed mice [106], suggesting that targeting 5-HT signaling represents a promising therapeutic strategy for ameliorating obesity-related glucose and lipid metabolic disorders.

The gut microbiota is vital to the systemic regulation of lipid digestion and absorption in the intestine via enteroendocrine signaling pathways [107]. Dysbiosis of the gut microbial ecosystem has been consistently linked to an elevated risk of metabolic disorders in both animal models and human populations [108]. A recent cohort study found that obesity was linked with an elevation in the *Prevotella*/*Bacteroides* ratio and a decrease in fecal tryptophan, a metabolite related to 5-HT biosynthesis [109]. 5-HT levels elevated the expression of several proteins involved in intestinal fatty acid absorption in vitro, and increased expression of those proteins was observed in HFD-fed mice monoassociated with *Clostridium ramosum* [110]. Genetic deficiency of the SERT also exacerbates HFD-induced metabolic disturbances and is associated with distinct gut microbial alterations, including *Intestinimonas*, *Atopostipes*, and *Erysipelatoclostridium*, genera implicated in serotonergic and lipid metabolism, thereby suggesting that SERT dysfunction may modulate obesity susceptibility through microbiota-mediated mechanisms [111]. In addition, it has been recently reported that the gut microbiota–5-HT signaling closely regulates HFD-induced obesity, where *Bacteroides vulgatus* blocks lipid absorption by inhibiting 5-HT-dependent transient receptor potential vanilloid 1 channel activation, providing a potential rationale for targeting the microbiota–5-HT signaling in metabolic disease therapy [15]. Additionally, *Lacticaseibacillus rhamnosus*-derived postbiotic ameliorates HFD-induced metabolic dysfunction by reshaping gut microbiota, reprogramming tryptophan metabolism to reduce colonic 5-HT, thereby restoring intestinal barrier integrity, suppressing inflammation, and improving glucose metabolism abnormalities [112]. These diverse observations collectively support a mechanistic framework in which the gut microbiota modulates intestinal 5-HT signaling to influence host metabolic homeostasis. Specific microbial taxa can shape intestinal 5-HT signaling, thereby affecting dietary fat absorption, chylomicron production, and systemic glucose metabolism. Notably, genetic ablation of serotonergic components or HFD-induced gut microbiota dysbiosis alters metabolic phenotypes in preclinical models of obesity. Conversely, microbiota-targeted interventions improve metabolic outcomes in association with the restoration of intestinal 5-HT levels. Collectively, these findings establish the gut microbiota–5-HT signaling as a critical regulator in obesity and related metabolites disorders.

Beyond 5-HT, other microbial metabolic pathways are also important in metabolic health. Notably, a prior report found that tryptamine, as bacterial tryptophan metabolites, can directly suppress fat mass in a HFD model [58]. In the context of HFD-induced metabolic dysfunction, gut microbiota-induced increased tryptamine is positively correlated with elevated systemic 5-HT levels, suggesting a potential functional connection between microbial tryptophan metabolism and host serotonergic signaling [113]. Similarly, HFD feeding alters the composition of gut microbiota via influencing the generation of bile acids. Treatment with gut commensal *Christensenella minuta* ameliorated metabolic dysfunction in HFD-induced obese mice, correlating with a significant elevation in 3-O-acylated bile acid levels [114]. *Akkermansia muciniphila* outer membrane protein ameliorates HFD-induced obesity and metabolic dysfunction by regulating bile acid synthesis via G protein-coupled bile acid receptor 1 signaling, thereby improving glucose and lipid homeostasis in mice [115]. Administration with *Bacteroides vulgatus*-metabolizing bile acid (e.g., cholic acid) mitigates HFD-induced obesity by inhibiting accumulation of lipid droplets and the retention of chyle particles in jejunal epithelial cells [15]. Importantly, bile acids signaling has been shown to modulate enteroendocrine cell activity, and emerging evidence suggests potential crosstalk with serotonergic pathways [116], although the precise mechanisms linking bile acid to 5-HT signaling remain to be fully understood. These findings indicate that gut microbiota modulates host metabolic homeostasis through a complex, interconnected pathway encompassing intestinal 5-HT signaling and multiple microbial-derived metabolites, whose crosstalk and hierarchical regulatory relationships remain to be clarified. 

### 5.2. Potential Links Between Tea Polyphenol, Gut Microbiota-Derived 5-HT Signaling and Metabolic Health

**Figure 2:**
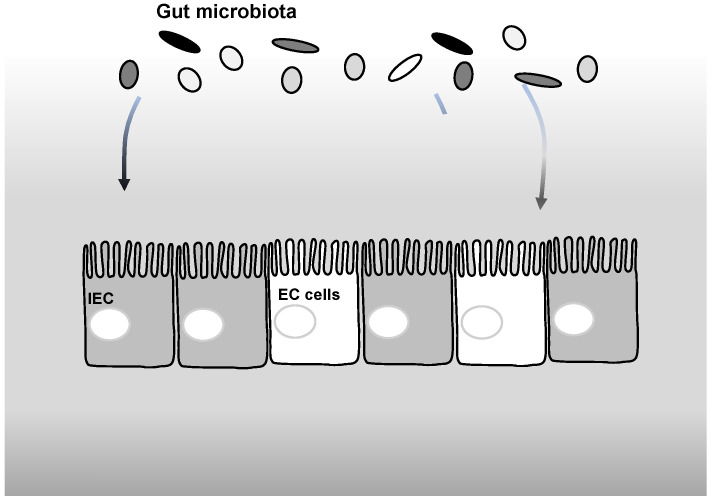
A proposed model of the interaction among tea polyphenols, gut microbiota, and 5-HT signaling in regulating obesity and metabolic health. Tea polyphenols (TPs) modulate the composition and metabolic function of the gut microbiota, contributing to increased production of metabolites such as short-chain fatty acids (SCFAs), bile acids, and indole derivatives. These microbial metabolites have been shown to directly stimulate host 5-HT biosynthesis and signaling. Although TPs, gut microbiota-derived metabolites, and 5-HT are each linked to the regulation of gut function and metabolic homeostasis, it remains unresolved whether TP-induced shifts in microbial metabolism exert their beneficial effects on obesity and related disorders specifically through modulation of the 5-HT pathway.

Emerging evidence from pre-clinical and clinical studies suggests a potential association between gut microbiota–host 5-HT signaling crosstalk and the beneficial effects of TPs on metabolic homeostasis (Figure 2). Rats trials suggested that TP addition is associated with altered 5-HT synthesis, alongside improved intestinal function [117,118]. EGCG administration consistently enriches beneficial genera such as *Dubosiella* and *Akkermansiae*, which are known producers of SCFAs. This shift in microbial composition is associated with enhanced SCFA synthesis and may contribute to the restoration of gut barrier function and alleviation of HFD-induced metabolic syndrome [119]. Given that SCFAs have been demonstrated to be well-established regulator of intestinal-derived 5-HT synthesis [13,53,54,77], it is therefore plausible that the TP-mediated increase in SCFA-producing bacteria may influence intestinal serotonergic signaling. Although evidence from metabolic disease models remains limited, studies in a constipation-predominant irritable bowel syndrome in a rat model have suggested that Mao Jian green tea extract can modulate colonic 5-HT availability, simultaneously enhance gut microbial diversity and enriches beneficial 5-HT-associated bacteria in the intestine, such as *Lactobacillus* [120], suggesting an indirect link between TP-based gut microbiota changes and intestinal 5-HT signaling. In addition, in human subjects, acute ingestion of TPs significantly increased systemic 5-HT levels alongside changes in microbial metabolites such as hippurate and salicylate [121]. More importantly, Theabrownin, a high-molecular-weight pigment derived from the oxidative polymerization of TPs during dark tea fermentation, demonstrates its anti-obesity effects by enriching 5-HT-associated bacterial taxa such as *Akkermansia*, *Bacteroides*, and *Parabacteroides*, enhancing fatty acid oxidation. However, these metabolic benefits are inhibited upon antibiotic-induced depletion of gut microbiota [122], providing direct experimental evidence that the gut microbiota is indispensable for theabrownin, potentially involving the microbiota–5-HT signaling. Collectively, these findings support that TPs may modulate intestinal serotonergic signaling via gut microbiota–host crosstalk, highlighting a plausible link between microbiota–5-HT signaling that underlies the beneficial effects of TPs on metabolic health maintenance.

Beyond SCFAs, TP also promotes the generation of gut microbial-derived metabolites such as indole derivatives [37,123]. Since indole derivatives, as potent stimulators, can directly promote human and mouse transient receptor potential ankyrin 1 and intestinal 5-HT secretion [14], the TP-induced elevation of these metabolites provides another plausible route through which TP may influence serotonergic signaling to regulate gut inflammation and oxidative stress, thereby improving metabolic outcomes. In mice, TP consumption was accompanied by the increasing production of bioactive metabolites (e.g., such as indole, indole-3-acetic acid, and 5-HT), and concurrent mitigation of intestinal inflammation and lipid metabolic dysfunction, suggesting a possible role of these metabolites in mediating TP effects [124]. In conclusion, TP intake correlates with changes in gut microbiota, microbial metabolites (e.g., indole derivatives), 5-HT levels, and metabolic and intestinal homeostasis. However, the causal interplay among these factors and the potential mediating role of 5-HT requires rigorous experimental validation.

Current evidence linking TP to metabolic health through the gut microbiota–5-HT pathway is predominantly indirect, with only a limited number of studies providing direct experimental validation. Clinically approved agents for metabolic disorders, including metformin, glucagon-like peptide-1 (GLP-1) receptor agonists, and insulin, exert therapeutic effects through well-defined molecular targets, quantifiable therapeutic effects, and are supported by robust clinical evidence. Specially, the widely used antidiabetic drug metformin has been shown in preclinical models to ameliorate HFD-induced metabolic dysfunction, partly by enriching beneficial gut microbiota (e.g., Faecalibacterium) and influencing intestinal 5-HT synthesis, and consequently effectively suppressing body weight gain and improving glucose homeostasis [125], highlighting potential relevance of microbial–serotonergic pathway in its action. Relevant mechanism studies found that chemical activation of 5-HTR4 elevates colonic GLP-1 level, upregulates GLP-1-encoding Gcg expression, and improves glucose tolerance in HFD-fed mice [126]. The selective 5-HTR4 antagonist GR113808 alleviates HFD-triggered body weight gain, inflammation, and glucose and triglyceride metabolic disorders, and prevents HFD-induced obesity in mice [106]. These seemingly paradoxical roles of 5-HTR4 in metabolic dysfunction can be attributed to the tissue- and cell-specific nature of 5-HT signaling. Of note, while clinically approved agents such as GLP-1 receptor agonists and metformin demonstrate robust metabolic benefits in patients with overweight and obesity, the effects of TP on the modulation of the microbial–serotonergic pathway remain modest and largely confined to limited preclinical models. In contrast to established pharmacotherapies with defined molecular targets and clinical validation, the putative involvement of the gut microbiota–5-HT signaling in TP actions lacks mechanistic clarity and human evidence, highlighting a critical knowledge gap. Such efforts could clarify whether the gut microbiota–5-HT signaling contributes to TP-associated metabolic benefits and, if so, inform the rational design of microbiota or 5-HT-targeted dietary strategies for metabolic disorders (Table 1).

## 6. Conclusions and Perspectives

In summary, TPs contribute to the maintenance of gut health and metabolic homeostasis through their interaction with the gut microbiota. TPs not only facilitate an improved microbial composition but are also transformed by microbes into various metabolites with enhanced bioactivity. These processes could collectively influence the gut microenvironment and potentially interact with the intestinal 5-HT signaling pathway. Given the critical role of 5-HT in gut function, the maintenance of 5-HT homeostasis is important to the beneficial effects of TPs on gut motility, barrier integrity and on the mitigation of obesity-related metabolic disturbances. A deeper understanding of how TP-modulated gut microbiota influence the context-dependent roles of 5-HT signaling in gut function and systemic metabolic homeostasis is needed to inform dietary approaches targeting gut microbiota–5-HT interactions in obesity and related metabolic diseases. Future studies could investigate whether microbial transformation products of TP, generated via in situ biotransformation in the gut, endogenously regulate 5-HT biosynthesis.

## Figures and Tables

**Table 1:** The effects of tea polyphenols on the gut microbiota and serotonin signaling systems ^1^.

Interventions	Model System	Microbial Changes	Metabolic Findings	Serotonin Signaling Changes	References
EGCG	HFD-induced obesity mice	Increased the abundance of *Dubosiella* and *Akkermansia*	Elevated SCFAs mitigated ileal barrier dysfunction and obesity	Unknown	[119]
Mao Jian green tea extract	Irritable bowel syndrome with constipation in a rat model	Increased the abundance of *Lactobacillus* sp. *Akkermansia*, *Bacteroides* and *Parabacteroides*.	Activated the calmodulin/myosin light chain kinase pathway	Increased SERT and decreased TPH1 expression in the colonic tissues	[120]
Theabrownin	HFD-fed mice	Increased the abundance of *Akkermansia*, *Bacteroides* and *Parabacteroides*	Reduced body weight gain and body fat rate	These genera were positively correlated to the level of 5-HT in blood circulation	[122]

^1^ Of the cited studies, only [120] provides direct evidence of gut-derived serotonergic modulation in a rat irritable bowel syndrome model. In contrast, the two metabolic studies [119,122] either omitted serotonergic assessments [119] or reported only correlative changes in circulating 5-HT levels without gut tissue analysis [122]. To date, it remains unverified whether microbiota-dependent regulation of the intestinal serotonin pathway by tea polyphenols occurs in the context of obesity or metabolic dysfunction. EGCG, epigallocatechin-3-gallate; HFD, high-fat diets; SCFAs, short-chain fatty acids; SERT, serotonin transporter; TPH1, tryptophan hydroxylase 1; 5-HT, serotonin.

## Data Availability

No new data were created or analyzed in this study.

## Abbreviations

ECs	Enterochromaffin cells
EGCG	Epigallocatechin-3-gallate
ENS	Enteric nervous system
FFARs	Free fatty acid receptors
GI tract	Gastrointestinal tract
GLP-1	Glucagon-like peptide-1
HFD	High-fat diets
5-HT	Serotonin
5-HTRs	Serotonin receptors
LPS	Lipopolysaccharide
NF-kB	Nuclear factor kappa-B
SCFAs	Short-chain fatty acids
SERT	Serotonin transporter
TP	Tea polyphenols
TLR4	Toll-like receptor 4
TPH1	Tryptophan hydroxylase1
TPH2	Tryptophan hydroxylase2
